# Assessment of Autoimmune Responses Associated with Asbestos Exposure in Libby, Montana, USA

**DOI:** 10.1289/ehp.7431

**Published:** 2004-09-30

**Authors:** Jean C. Pfau, Jami J. Sentissi, Greg Weller, Elizabeth A. Putnam

**Affiliations:** Center for Environmental Health Sciences, Department of Biomedical and Pharmaceutical Sciences, University of Montana, Missoula, Montana, USA

**Keywords:** asbestos, ANA, environmental autoimmunity, immunotoxicology

## Abstract

Systemic autoimmune responses are associated with certain environmental exposures, including crystalline particles such as silica. Positive antinuclear antibody (ANA) tests have been reported in small cohorts exposed to asbestos, but many questions remain regarding the prevalence, pattern, and significance of autoantibodies associated with asbestos exposures. The population in Libby, Montana, provides a unique opportunity for such a study because of both occupational and environmental exposures that have occurred as a result of the mining of asbestos-contaminated vermiculite near the community. As part of a multifaceted assessment of the impact of asbestos exposures on this population, this study explored the possibility of exacerbated autoimmune responses. Age- and sex-matched sets of 50 serum samples from Libby and Missoula, Montana (unexposed), were tested for ANA on HEp-2 cells using indirect immunofluorescence. Data included frequency of positive tests, ANA titers, staining patterns, and scored fluorescence intensity, all against known controls. Serum immunoglobulin A (IgA), rheumatoid factor, and antibodies to extractable nuclear antigen (ENA) were also tested. The Libby samples showed significantly higher frequency of positive ANA and ENA tests, increased mean fluorescence intensity and titers of the ANAs, and higher serum IgA, compared with Missoula samples. In the Libby samples, positive correlations were found between ANA titers and both lung disease severity and extent of exposure. The results support the hypothesis that asbestos exposure is associated with autoimmune responses and suggests that a relationship exists between those responses and asbestos-related disease processes.

Asbestos-related lung disease (ARD), including fibrosis, pleural plaques, and cancer, continues to be a serious and significant problem despite increasing awareness of the health hazards of asbestos inhalation. Although the exact mechanisms leading to the progression of these conditions have not been fully explained, there is evidence that some of the lung pathologies seen with both asbestos and silica exposures are immunologically mediated (Hamilton et al. 1996; Holian et al. 1997; Perkins et al. 1993). Silica and asbestos exposures also both appear to exacerbate autoimmune responses. Epidemiologic studies have shown strong associations between silica exposure and several autoimmune diseases, including scleroderma, systemic lupus erythematosus (SLE), and rheumatoid arthritis (RA) (Koeger et al. 1995; Parks et al. 1999; Powell et al. 1999; Steenland and Goldsmith 1995). Increased serum immunoglobulins, positive antinuclear antibody (ANA) tests, and immune complexes have been reported in small cohorts of individuals exposed to asbestos (Lange 1980; Nigam et al. 1993; Zerva et al. 1989), but to our knowledge no comprehensive study has been undertaken to assess the prevalence, specificity, and significance of autoantibodies associated with asbestos exposures. The population in Libby provides a unique research opportunity because of significant exposures that occurred as a result of the mining of asbestos-contaminated vermiculite near the community. Exposures have been documented not only in the miners but also in their family members, as well as anyone who used the vermiculite or played near the mine tailings. Therefore, the Libby asbestos exposures were both occupational and environmental throughout the community (Peipins et al. 2003).

In addition to the ARD in Libby, there have been anecdotal reports of an increased prevalence of systemic autoimmune disease (SAID), but verification of these diagnoses is still in progress. When the Centers for Disease Control and Prevention/Agency for Toxic Substances and Disease Registry (ATSDR) performed its screening in Libby during 2000–2001, 494 (6.7%) of 7,307 screening participants indicated that they had been diagnosed with either SLE, scleroderma, or RA (Larson T, personal communication). In contrast, a prevalence of < 1% for these three conditions combined would be expected based on pooled estimates from 43 prevalence studies (Jacobson et al. 1997). These data, along with extensive evidence of silica-associated auto-immunity, provided the impetus to initiate a multifaceted assessment of the impact of asbestos exposures on the population of Libby, Montana, including possible autoimmune responses.

In this article we report on a study designed to assess whether there were humoral alterations in serum samples from an asbestos-exposed population (Libby samples) that might indicate autoimmune responses, providing the rationale for future full-scale studies. Serum samples of subjects from Libby and from a Montana community with no reported asbestos exposure were assayed for a variety of immune parameters, including ANA, immunoglobulin A (IgA), rheumatoid factor (RF), and antibodies to extractable nuclear antigens (ENA).

## Materials and Methods

### Human samples.

All samples were acquired according to approved University of Montana institutional review board protocols, protecting the well-being and confidentiality of all subjects. Appropriate informed consent was obtained from all subjects, and a questionnaire was administered regarding overall health, smoking status, asbestos exposure, age, sex, and recontact information.

Two sample pools were obtained through other studies at the Center for Environmental Health Sciences. Subjects were recruited through flyers and ads in Missoula, Montana, for a study of immune function (E. Putnam), and serum samples were collected at the same time for convenience. Missoula is similar to Libby in that it is located in a mountain valley subject to similar climatic conditions, including winter inversions, dry summers, and exposure to smoke from fall forest fires. Because prevailing winds in Missoula are from the west and Libby is well to the north, there would be no transfer of asbestos from the Libby airshed to the Missoula airshed. Therefore, on a relative basis, although one cannot exclude the possibility that there could have been some minimal asbestos exposure by the Missoula population, it is an acceptable reference population for the Libby subjects who were definitely exposed to asbestos. For this study, we selected 50 samples from subjects with no reported asbestos exposure from the Missoula pool, excluding any who had lived or worked in Libby. Concurrently, subjects were being recruited from Libby, Montana, for a genetic study of ARD susceptibility (E. Putnam), and the samples were drawn at the Center for Asbestos Related Diseases Clinic in Libby when subjects came for screening or responded to subject recruitment advertisements. From this pool, 50 Libby subjects were selected that were matched to the 50 Missoula subjects to give similar mean age and sex ratios for the two subject sets. Permission was obtained to acquire information about prescription drug use and ARD status from medical records, and these data were inserted into a coded database.

Both communities from which subjects were drawn are fairly homogeneous in terms of ethnicity; most residents are of northern European descent, with 94.7% white in Lincoln County (Libby) and 93.6% white in Missoula County (Missoula) according to Montana census data (U.S. Census Bureau 2004). The mean age for both sample sets was 55 years (Missoula, 54.8 ± 2.5 years; Libby, 55.0 ± 2.1 years), and the male:female ratio was 25:25 for both sets. An exclusion criterion was the use of medications strongly associated with drug-induced autoimmunity (Fritzler 1994). The presence of diagnosed autoimmune disease did not exclude individuals from either sample set but was noted on intake. None of the Missoula subjects recruited had diagnosed SAID. Of the Libby subjects recruited initially for the genetics study, the percentage with diagnosed SAID was 5.9%, and in the final Libby sample set of 50, there were two with SLE, 1 with multiple sclerosis, and 1 with RA. Because the individual with RA also had SLE, this means that three people in 50 had SAID (6%). These values are consistent with the ATSDR screening data for this community (Larson T, personal communication).

The lung diseases in this community have been previously described (Peipins et al. 2003) and include primarily pleural abnormalities (17.8%) and interstitial abnormalities (< 1%). In our Libby sample set, 12 (24%) had no reported abnormalities, 27 (54%) had pleural abnormalities, 8 (16%) had interstitial abnormalities, and 3 (6%) had a combination of pleural and interstitial abnormalities.

### Sample and data collection.

The blood samples were collected, and serum samples were obtained and frozen by standardized clinical methods to prevent differences due to handling. The samples were blinded with only sex and age noted, and stored at −80°C until assayed. After testing, coded information regarding disease status and exposure was obtained from the questionnaire and ATSDR screening data (ATSDR 2002).

### ARD and asbestos exposure rankings.

ARD status, based on data recorded in the database primarily as a result of the ATSDR screenings, was ranked on a scale of 0–3, as described in Table 1. The rankings were intentionally simplified, based on radiographic evidence of single versus extensive plaques or interstitial abnormalities, as well as spirometry evidence of functional deficits. For example, a subject with a single pleural plaque and no functional deficit would be scored at 1, whereas a subject with bilateral plaques and effects on spirometry was scored at 3. To further break down the sample sets by disease types would have made the subsets too small for statistical analysis. Exposure status was ranked on a scale of 0–4, as described in Table 2. These scores were also simplified in order to focus on duration of exposure and the existence of occupational and/or environmental exposure to asbestos. The rankings of the Libby subjects were performed independently by two of the researchers (J.C.P. and E.A.P.).

### Autoantibody testing.

A clinical test for nuclear antigens (ANA assay), used to screen for antibodies commonly seen in SAID, was performed at a screening dilution of the sera. All serum samples were diluted 1:40 in phosphate-buffered saline (PBS) and tested by indirect immunofluorescence (IIF) on a single lot of commercially prepared and fixed HEp-2 cells (ImmunoConcepts Inc., Sacramento, CA), according to manufacturer’s instructions. The staining pattern and relative fluorescence intensity were compared with known positive and negative controls using a Zeiss fluorescence microscope with 40× objective and recorded as positive (1+ to 4+) or negative (0). The staining pattern was also noted and recorded. The same microscope and settings were used for all samples, and the slides were read by two independent readers. Samples showing homogeneous staining patterns were reevaluated using the *Crithidia luciliae* substrate (ImmunoConcepts), which specifically detects antibodies to double-stranded DNA (dsDNA), and by enzyme-linked immunosorbent assay (ELISA) to detect antibodies to chromatin (INOVA Diagnostics, San Diego, CA), both according to the manufacturers’ instructions. Samples with positive ANAs were also evaluated using a modified ANA test to determine whether any of the anti-ANA antibodies were of the IgA isotype. The samples were tested on HEp-2 slides as above, but instead of the anti-human IgG fluorescein isothiocyanate (FITC) conjugate included with the slides, we used goat anti-human IgA FITC conjugate (Southern Biotech, Birmingham, AL). The slides were read as described above.

### IgA ELISA.

For detection of serum IgA, the mucosal antibody isotype, 96-well polysorb plates (Nunc, Rochester, NY) were coated with 1 μg/mL of anti-human kappa light chain (Southern Biotech) in carbonate coating buffer overnight at 4°C. Wells were then blocked with PBS containing 1% bovine serum albumin (BSA) for 1 hr. Subject samples were diluted 1:4,000, 1:8,000, and 1:16,000 in diluent buffer (PBS, 1% BSA, 0.1% Tween-20). Samples, standards, and blanks were added to wells to give 100 μL/well. After 1 hr, plates were washed with three changes of PBS containing 0.1% Tween-20. The detection antibody [goat anti-human IgA alpha chain coupled to horseradish peroxidase (HRP); Caltag, Burlingame, CA] was added and the plates were incubated for 1 hr at room temperature. The plates were washed again and developed using HRP-tetramethylbenzidine (TMB) substrate (Zymed, San Francisco, CA). The reaction was stopped with 2N H_2_SO_4_, and the plate was read on a SpectraMax plate reader (Molecular Devices, Sunnyvale, CA). All data were evaluated against a standard curve, using human IgA (Sigma, St. Louis, MO).

### RF ELISA.

RF in the subjects’ serum was measured with an ELISA kit according to the manufacturer’s protocol (INOVA Diagnostics). The plates were read on the SpectraMax plate reader. Optical density (OD) values were compared with known controls provided with the kit and rated as negative or positive (marginal, moderate, or high).

### ENA array.

Analyses of antibodies to five extractable nuclear proteins commonly seen in SAID (Sm, RNP, SS-A, SS-B, and Scl-70) were performed using an addressable bead array kit (QuantaPlex ENA-5; INOVA Diagnostics) according to the manufacturer’s instructions, on a Luminex multiplex system (MiraiBio, Alameda, CA). The values were compared by using MasterPlex software (MiraiBio) to negative and graduated positive control reagents provided with the kit, and determined to be low, moderate, or high positive, or negative.

### Statistics.

In this study we included analysis of several different types of data, including percentages/frequencies (e.g., ANA frequencies), ordinal (e.g., disease status assessed on a 4-point scale), and scale (e.g., mg/mL IgA) data. Consequently, we used the following statistical methods: *a*) differences in percentages were tested using raw frequencies with Fisher’s exact test; *b*) contingency tables with 4- and 5-point ordinal level frequency comparisons were made via the chi-square test; and *c*) independent sample *t*-tests were used for scale measures. In the Libby samples, comparisons (correlations) between ANA levels and disease and exposure rankings were made using the nonparametric Spearman rank correlation. In all analyses we used two-tailed, unpaired analyses, and reported 0.05 type I error levels. Data reported in the text are mean ± SEM.

## Results

### Frequency and fluorescence intensity of positive ANAs.

**Figure 1:**
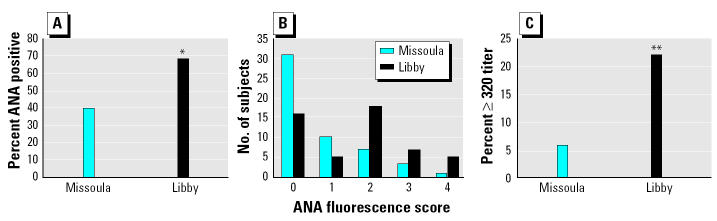
Comparison of ANA in Libby samples with the Missoula samples. (*A*) Positive and negative tests determined based on known controls provided with the kits (*p = 0.004, Fisher’s exact test; *n* = 50). (*B*) ANA fluorescence intensity score based on known controls (*p* = 0.004, Pearson chi-square test). (*C*) Percentage in each group with a titer > 320 (**p < 0.01, Fisher’s exact test; *n* = 50). ANA tests were performed as described in “Materials and Methods.” Groups were screened at a serum dilution of 1:40 and read by two readers; positive tests were then titered to 1:1,280.

The 50 serum samples in each set were tested by IIF and were determined to be positive or negative for ANA based on known controls. Figure 1A shows that the relative frequency of positive ANAs was 28.6% higher in the Libby sample set than in the Missoula set (*p* = 0.006). Because low-titer positive ANAs are fairly common in normal populations, the ANA slides were scored for fluorescence intensity. The scored mean fluorescence intensity of positive ANAs, rated on a scale of 1–4 against known controls, was higher in the Libby sample set (mean = 2.34 ± 0.153) compared with those from Missoula (1.76 ± 0.194; *p* = 0.02), and the distribution of subjects receiving the various scores was shown to be significantly different between the sample sets (*p* = 0.004; Figure 1B). The scored fluorescence intensity is not a direct quantification of autoantibodies but generally suggests a higher titer. Therefore, the positive samples were subsequently titered to 1:1,280 and further analyzed for ANA. In both groups, the ANA scores and titers were highly correlated, suggesting that the scoring provides a close estimation of titer (Missoula: correlation coefficient = 0.502, *p* < 0.001; Libby: correlation coefficient = 0.828, *p* < 0.001; Table 3). We found that the distribution of titers for samples in the two sample sets were significantly different (*p* = 0.036; data not shown). The percentage of subjects having a positive ANA at a titer ≥320 is shown in Figure 1C, again showing a significant difference between the two sample sets.

### IgA levels and RF.

**Figure 2:**
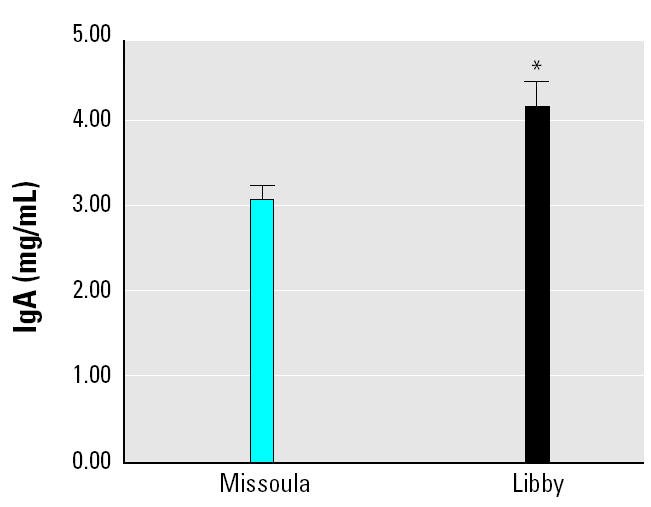
Serum IgA levels were significantly higher in the Libby group. Serum IgA was measured by ELISA using anti-human IgA capture and detection (HRP-conjugated) antibodies from Southern Biotech and Caltag, respectively. The samples were tested in duplicate, developed using TMB, and read on a SpectraMax plate reader. OD was compared with a standard curve (human IgA, Sigma) to calculate values (mg/mL, *n* = 50).
*p = 0.002, two-tailed *t*-test.

Because other studies of asbestos-exposed populations have shown differences in serum IgA compared with unexposed subjects, serum IgA levels were analyzed by ELISA in our sample sets. The Libby samples showed significantly higher levels of serum IgA than the Missoula samples (Figure 2). Although both sample sets had mean IgA concentrations within normal ranges (~ 0.9–4.5 mg/mL), the Libby mean was at the high end of the range (4.2 mg/mL). The ANA tests were subsequently modified to detect IgA rather than IgG, and all of the ANA tests were negative in both sample sets, indicating that the autoantibodies were most likely primarily IgG and not IgA (data not shown).

**Figure 3:**
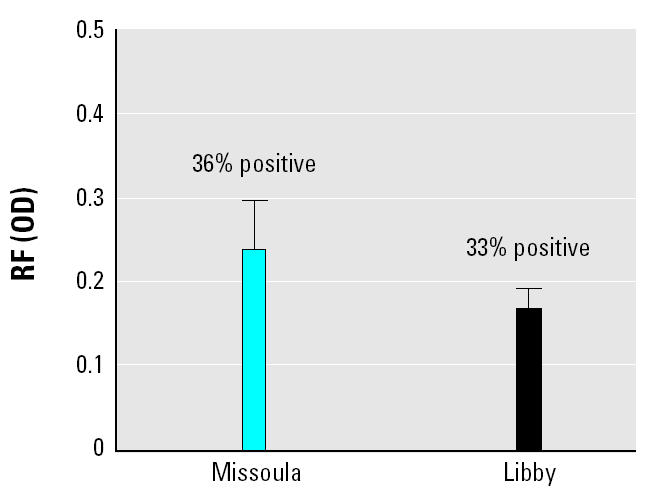
Evaluation for IgM RF by ELISA. Neither the percent positive (based on kit positive and negative controls) nor the mean OD calculated for each group was statistically significant (Fisher’s exact test and *t*-test, respectively).

RF consists of IgM or IgG antibodies directed against the constant domain of immunoglobulin. These autoantibodies can lead to immune complex deposition in tissues and are associated with a variety of infectious and inflammatory disorders such as RA. The samples were evaluated for IgM RF by ELISA, and there were no differences in either the mean OD calculated for each sample set or the frequency of positive tests for RF (Figure 3).

### Staining patterns on ANAs.

**Figure 4:**
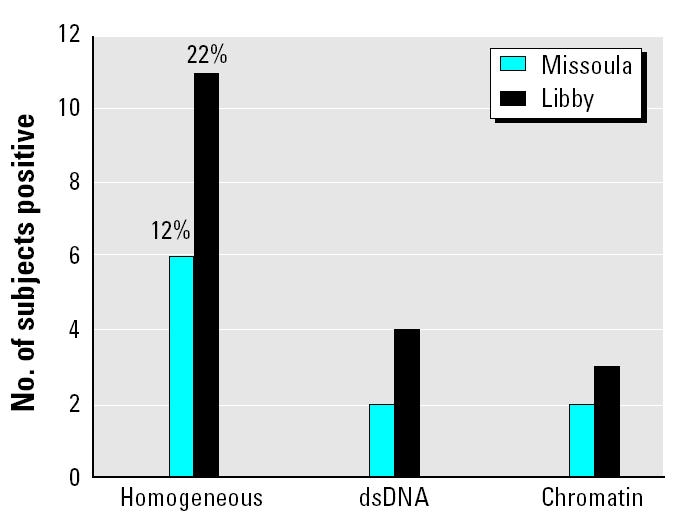
Assessment of autoantibodies targeted to dsDNA or chromatin in subjects with homogeneous ANA patterns. See “Materials and Methods” for details. Tests for antibodies to individual histones were not performed, but that target is indicated for those homogeneous patterns without antibodies to dsDNA or chromatin itself.

To determine the specific targets for the IgG autoantibodies, we analyzed the patterns visible on the ANA tests. In the Missoula samples, a nuclear speckled staining pattern was most common, as expected in a normal population (speckled, 14%; homogeneous, 12%; nucleolar, 10%). Other staining patterns were relatively rare, as expected. However, in the Libby samples, homogeneous (indicative of antibodies to chromatin) and nucleolar staining patterns were more prevalent, although the differences were not statistically significant (speckled, 18%; homogeneous, 22%; nucleolar, 18%). We tested for specific antibodies related to these IIF patterns using an ELISA for chromatin and an IIF assay for anti-dsDNA (*Crithidia luciliae* test). The results indicated that although 22% of the Libby group had homogeneous staining patterns, less than half of those were positive for either chromatin or dsDNA (Figure 4). This suggests that either individual histones not available for binding in chromatin preparations (i.e., H3, H4) or other components of chromatin may be the targets being recognized by the autoantibodies in these individuals.

### Serum antibodies to ENAs.

**Figure 5:**
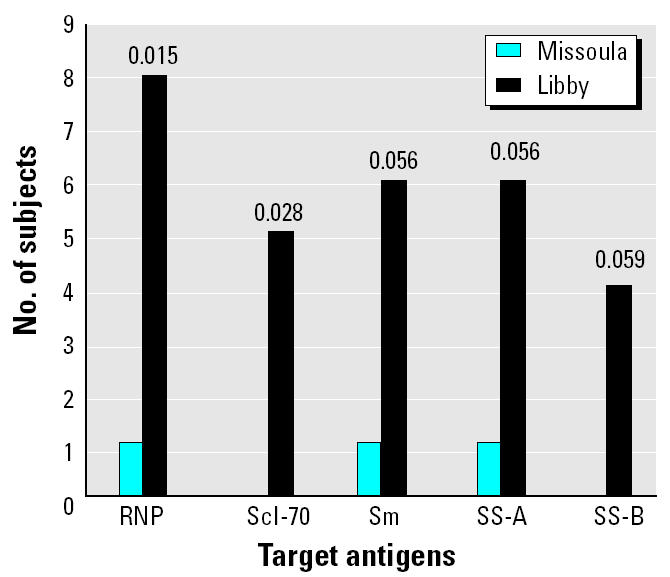
Increased frequency of positive ENA tests in Libby samples compared with Missoula samples. Tests were performed as described in “Materials and Methods.” Numbers above the bars are *p*-values indicating the statistical difference between Libby samples and Missoula samples (determined by Fisher’s exact test; *n* = 50).

To further explore possible targets for the autoantibodies, we used a Lumine multiplex analyzer with an addressable laser bead immunoassay to detect antibodies to five ENAs. Twelve of the Libby samples (24%) had positive ENA tests, with most of the positive samples having more than one of the antibody specificities. The Missoula sample set had only two samples (4%) with positive ENA tests. These differences were statistically significant by Fisher’s exact test (*p* = 0.004; data not shown). Figure 5 shows the distribution of positive tests in each group for all five antigens tested.

### Immune parameter correlations.

Table 3 shows statistical correlations among tested immune parameters within the Libby sample set, using Spearman’s rho nonparametric test. As might be expected based on the epidemiology of autoimmune diseases, the age of the individual was positively correlated with the ANA titer, ANA score, and RF. RF was also correlated with ANA titer. IgA levels were not correlated with any other parameter, so the physiologic significance of the elevation seen in the Libby samples remains unclear.

### Correlation of assay results with extent of exposure and ARD.

In addition to the correlations shown above, the immune parameters were analyzed against the scores of exposure levels and ARD status, as described in “Materials and Methods.” Table 4 shows that there were significant positive associations between ANA titers and both asbestos exposure and disease status. Because a central hypothesis relating to asbestos toxicology is that asbestos exposure is positively associated with ARD, we tested that correlation using our scoring system. The correlation between disease and exposure was 0.239, consistent with the hypothesis (Table 4).

**Figure 6:**
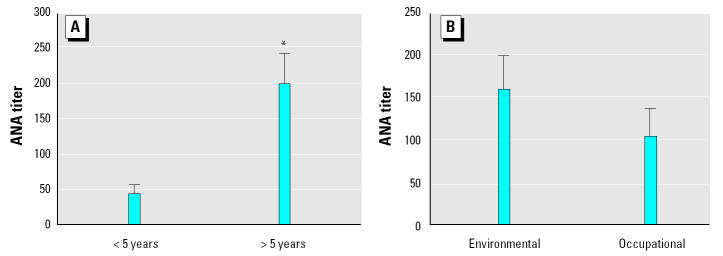
ANA titer (mean ± SD) for subsets of the Libby samples based on length of asbestos exposure (*A*) or source of exposure (*B*) as environmental (household contact, used in garden, etc.) or occupational (worked in vermiculite mine or processing).
*p < 0.01 by two-tailed *t*-test.

The analysis for asbestos exposure was performed using the graded system described in Table 2, but in looking at the data, it was apparent that the largest effect on ANA titer was seen in terms of length of exposure rather than the source (occupational or environmental). Figure 6A shows the mean ANA titers for the Libby samples when subgrouped by duration of exposure and demonstrates that the titers were significantly higher for those subjects exposed to asbestos for > 5 years. When samples were separated according to whether the subject was exposed environmentally or occupationally, no significant difference in mean ANA titer was observed in those two groups (Figure 6B). This suggests that the duration of exposure had a greater impact on autoimmune responses than the source of exposure. It should be noted that none of the subjects reported solely occupational exposures; all also had environmental exposures as well.

## Discussion

By demonstrating an association between asbestos exposure and measures of auto-immune responses, this study supports and augments other existing evidence that, like silica, asbestos is an agent of systemic auto-immunity. Asbestos-contaminated vermiculite from Libby has been shipped and processed in many sites in the United States, and this material is still used in many applications. It therefore remains a significant health risk to humans both occupationally and environmentally, and an awareness of an association with autoimmunity could impact necessary monitoring, testing, and treatment regimens for exposed individuals or populations.

In addition, this study establishes the Libby population as an excellent cohort for further study of the immunologic aspects of asbestos toxicology. It is a unique population with both occupational and environmental exposures, excellent ongoing monitoring and demographic data, enthusiasm for participation in these studies, and sufficient numbers of exposed individuals to develop sample sets with adequate power for statistical analyses of many parameters. The objective of this study was to compare the frequency of serum auto-antibodies in two matched populations, one of them having significant asbestos exposure. It was designed as an initial study in order to explore the feasibility and justification for a more extensive study of autoimmunity in the Libby population.

Previous studies have measured several immune parameters in populations exposed, primarily occupationally, to asbestos. Nigam et al. (1993) demonstrated increased IgG and IgA and positive ANAs in asbestos-exposed individuals compared with controls, even in the absence of apparent ARD. This finding suggested that immune alterations may precede the onset of ARD. A high frequency of positive ANAs was also found in a Japanese group of asbestos plant workers (Tamura et al. 1993). Interestingly, a 3-year follow-up study of the Japanese group showed significant correlation of positive ANAs with progression of the disease, leading to additional diagnoses of asbestosis in a previously healthy group (Tamura et al. 1996); this suggests that auto-immunity may play a role in ARD. Because that important observation needs to be confirmed, the present study also forms the basis for a similar progressive study of the Libby population to explore the temporal relationship between autoimmunity and ARD. If autoimmune responses play a role in the progression of ARD, this would become an important target for therapeutic strategies.

A higher frequency of positive ANA was seen in the Libby sample set compared with the samples from Missoula, even though the Missoula ANA positive frequency was fairly high (40%). This high “normal” frequency may be due to the age of the population, the low dilution used (1:40) for the screening, or other unidentified population considerations. Nevertheless, the Libby sample set showed a 28.6% increase above that seen for the Missoula samples. In addition, the mean fluorescence intensity of the ANAs, as well as the titers, were higher in the Libby samples compared with the Missoula samples. Although higher titers of autoantibodies are not necessarily correlated with an autoimmune disease process, in some cases increased titers can indicate an exacerbation, relapse, or stage of an autoimmune process.

Other immune parameters showed differences in the Libby group as well, including increases in serum IgA. Serum IgA is generally found at relatively low levels, but increased levels are associated with some chronic inflammatory disorders, such as occupational lung disease, psoriatic arthritis, Crohn’s disease, and ankylosing spondylitis (Hoffman et al. 2003; Lindqvist et al. 2002; Zhestkov 2000). The significance of increased serum IgA levels in the Libby samples is not clear, but it is consistent with other studies of asbestos-exposed subjects (Nigam et al. 1993; Zerva et al. 1989). We demonstrated that the autoantibodies detected by ANA were not of the IgA isotype, so it is possible that the IgA antibodies are simply elicited by nonspecific chronic inflammatory processes in these individuals. This possibility is supported by the lack of correlation between IgA titers and either ANA or asbestos exposure; however, a correlation between IgA titers and disease status was also lacking in our analysis.

We found no difference between the two sample sets in terms of frequency of positive tests for RF, and in general the positive samples in both groups were rated marginal to moderate when compared with known controls (data not shown). There was also no correlation between RF and asbestos exposure. These results suggest that asbestos exposure is not associated with increases in RF, especially because previous studies of asbestos-exposed populations have not consistently reported elevated RF. Interestingly, we found a positive association of RF with lung disease status. This may suggest that the presence of RF is more dependent on the chronic inflammation resulting from the ARD than on asbestos exposure itself. Alternatively, because there were positive correlations between RF and both ANA titer and ARD, the threshold of asbestos exposure impacting development of RF may simply be too low to be detected using the simplified exposure scale (Table 2). Therefore, the pathophysiologic significance of these results remains to be determined with further study. Although assaying RF is a good screening tool, it is not specific for RA. An alternative test would be an assay for cyclic citrullinated peptide (CCP), a more specific epitope characteristic of autoantibodies in RA (Bombardieri et al. 2004; Saraux et al. 2003) because CCP can antedate clinical RA.

The Libby sample set had a significantly higher number of total positive ENA subjects (24% vs. 4% in the Missoula group). ENAs are defined target antigens in a variety of autoimmune diseases, including systemic lupus, mixed connective tissue disease, systemic sclerosis, and polymyositis (Pahor et al. 1998). In HEp-2 cells, most of these antibodies produce a speckled or atypical speckled pattern. Therefore, our combined data suggest that the major responses in asbestos-exposed individuals include antibody development to targets such as chromatin components (i.e., histone) or nucleolar components (fibrillarin, DNA topo-isomerase I), which give the homogeneous and nucleolar patterns, and several ENAs that give the speckled pattern. There are auto-antibodies—other than those detected by the assay used here—that could give the speckled patterns seen in both populations. Analysis of a more comprehensive array of autoantigens may provide more insight into the spectrum of autoantibodies related to asbestos exposure.

The presence of autoantibodies does not necessarily suggest a disease process. However, to begin to explore a possible role of these antibodies in the health of the population in Libby, data regarding the extent of ARD severity were gathered as described in “Materials and Methods.” Extent of exposure included both duration and site of exposure (work vs. recreational/home). The data showed that individuals with longer exposures, and especially if exposed both at work and at home, had higher ANA titer than those with shorter exposures (< 5 years). Disease status considered the extent of fibrosis or plaquing, as well as functional deficits. Again, the data showed increasing ANA titer with increasing disease severity. The stronger correlation between ANA titer and disease than between ANA titer and exposure may be due to imprecise scoring systems. Exposure and ARD are assumed to be correlated (Peipins et al. 2003), even though this correlation was only at the 90th percentile using our scoring systems. Therefore, these data do not exclude the possibility that the correlation of ANA titer with exposure may be secondary to the chronic inflammation and tissue damage of the associated ARD. It is interesting to note, however, that of those 494 participants from the Libby Tremolite Asbestos Registry cohort with suspected SAID, 171 (35%) have had pleural and/or interstitial abnormalities indicated on chest radiographs and confirmed by two B-readers (Larson T, personal communication). These cross-sectional findings suggest that the proportion of radiographic abnormalities among participants with suspected SAID (35%) was almost double the proportion of radiographic abnormalities among the entire Libby cohort (~ 18%) (Peipins et al. 2003). Further study is required to determine if, and how, autoimmune responses are related to ARD and whether autoimmunity influences ARD progression.

## Conclusion

We have established that there are significant differences in frequency and titer of positive ANA tests, frequency of positive ENA tests, and higher levels of serum IgA when an asbestos-exposed Libby cohort was compared with one from Missoula with no reported asbestos exposures. We have also shown significant associations between asbestos exposure and ANA titer. These data support the hypothesis that asbestos exposure is associated with autoimmune responses. The correlations of ARD with ANA titer, ANA score, and RF suggest the possibility that auto-immunity could play a role in the progression of ARD. This study provides the foundation and justification for a larger and more extensive study, planned to explore these associations much more rigorously. These studies will increase our understanding of the immune components of ARD and could lead to improved interventions as an ultimate goal of the discovery of interrelated pathologies.

## Figures and Tables

**Table 1:** Simple classification of ARD severity.

Disease severity	Criteria used	Ordinal value
None	No reported lung pathology	0
Limited	Unilateral radiograph abnormality	1
Moderate	Bilateral abnormality, minimal functional deficit	2
Severe	Bilateral abnormality, severe or progressive functional deficit	3

**Table 2:** Asbestos exposure scores determined from screening data.

Asbestos exposure	Criteria used	Ordinal value
None	No reported occupational or environmental asbestos exposure	0
Minimal	< 5 years, only occupational or environmental	1
Low	< 5 years, both occupational and environmental	2
Moderate	> 5 years, only occupational or environmental	3
High	> 5 years, both occupational and environmental	4

**Table 3:** Correlation coefficients (CC) with immune parameters in the Libby cohort.

Parameter analyzed	Age	ANA titer
ANA score		
CC	0.399**	0.828**
Significance (two-tailed)	0.004	0.00
No.	51	50
ANA titer		
CC	0.381**	1.00
Significance (two-tailed)	0.006	
No.	50	
IgA (mg/mL)		
CC	−0.009	−0.278
Significance (two-tailed)	0.953	0.075
No.	43	42
RF (OD)		
CC	0.331*	0.351*
Significance (two-tailed)	0.030	0.023
No.	43	42

* Correlation is significant at the 0.05 level (two-tailed Spearman’s rho test).

** Correlation is significant at the 0.01 level (two-tailed Spearman’s rho test).

**Table 4:** Correlation coefficients (CC) between immune parameters and scores both of asbestos exposure and of asbestos-related disease.

Parameter analyzed	Exposure	ARD
Age		
CC	−0.015	0.304*
Significance (two-tailed)	0.920	0.034
No.	51	49
ANA score		
CC	0.252	0.295*
Significance (two-tailed)	0.074	0.040
No.	51	49
ANA titer		
CC	0.366**	0.392**
Significance (two-tailed)	0.009	0.006
No.	50	48
RF (OD)		
CC	0.129	0.388*
Significance (two-tailed)	0.410	0.010
No.	43	43
Exposure		
CC	1.00	0.239
Significance (two-tailed)		0.098
No.		49
IgA		
CC	−0.167	−0.090
Significance (two-tailed)	0.283	0.567
No.	43	43

* Correlation is significant at 0.05 (two-tailed Spearman’s rho test).

** Correlation is significant at 0.01 (two-tailed Spearman’s rho test).
